# Comparative proteomic analysis on chloroplast proteins provides new insights into the effects of low temperature in sugar beet

**DOI:** 10.1186/s40529-022-00349-6

**Published:** 2022-06-07

**Authors:** Jiali Long, Wang Xing, Yuguang Wang, Zedong Wu, Wenjing Li, Yi Zou, Jiaping Sun, Fushun Zhang, Zhi Pi

**Affiliations:** 1 School of Life Sciences, Heilongjiang University, Harbin, 150080 Heilongjiang China; 2 College of Advanced Agriculture and Ecological Environment, Heilongjiang University, Harbin, 150080 Heilongjiang China

**Keywords:** Sugar beet, Low temperature stress, Chloroplast, Proteome

## Abstract

**Background:**

Low temperature, which is one of the main environmental factors that limits geographical distribution and sucrose yield, is a common abiotic stress during the growth and development of sugar beet. As a regulatory hub of plant response to abiotic stress, activity in the chloroplasts is related to many molecular and physiological processes, particularly in response to low temperature stress.

**Results:**

The contents of chlorophyll (Chl) and malondialdehyde (MDA), relative electrical conductivity (REL), and superoxide dismutase (SOD) activity were measured. The results showed that sugar beet could manage low temperature stress by regulating the levels of Chl, REL and MDA, and the activity of SOD. The physiological responses indicated that sugar beets respond positively to low temperature treatments and are not significantly damaged. Moreover, to determine the precise time to response low temperature in sugar beet, well-known abiotic stresses-responsive transcript factor family, namely DEHYDRATION RESPONSIVE ELEMENT BINDING PROTEIN (*DREB*), was selected as the marker gene. The results of phylogenetic analyses showed that *BvDREBA1* and *BvDREBA4* were in the same branch as the cold- and drought-responsive *AtDREB* gene. In addition, the expression of *BvDREB*s reached its maximum level at 24 h after low temperature by RNA-Seq and qRT-PCR analysis. Furthermore, the changes in chloroplast proteome after low temperature at 24 h were detected using a label-free technique. A total of 416 differentially expressed proteins were identified. GO enrichment analysis showed that 16 GO terms were significantly enriched, particularly chloroplast stroma, chloroplast envelope, and chloroplast thylakoid membrane. It is notable that the transport of photosynthetic proteins (BvLTD and BvTOC100), the formation of starch granules (BvPU1, BvISA3, and BvGWD3) and the scavenging of reactive oxygen species (BvCu/Zn-SOD, BvCAT, BvPrx, and BvTrx) were the pathways used by sugar beets to respond to low temperatures at an early stage.

**Conclusions:**

These results provide a preliminarily analysis of how chloroplasts of sugar beet respond to low temperature stress at the translational level and provide a theoretical basis for breeding low temperature resistant varieties of sugar beet.

**Supplementary Information:**

The online version contains supplementary material available at 10.1186/s40529-022-00349-6.

## Background

Sugar beet (*Beta vulgaris* L.) is one of the most important sugar crops in the world, accounting for approximately 30% of global sugar production (Mutasa-Gottgens et al. 2012; Porcel et al. 2018; Xing et al. 2020). In China, the main planting areas are concentrated in three regions, namely Inner Mongolia, Xinjiang and Heilongjiang. In these areas, low temperature is a common abiotic stress in plant growth and development, which is one of the main environmental factors that limits crop yield and geographical distribution (Liu et al. 2019). During the vegetation period, the production of sugar beet is limited by late-spring frost and early autumn snow (Kaya and Kulan 2020). In particular, when seeds or seedlings were exposed to freezing temperatures at early developmental stages, the germination rate, survival rate and sucrose yield are severely restricted (Moliterni et al. 2015). Therefore, it is essential for sugar beet breeding to study the regulation of low temperature in its growth and development and improve tolerance to low temperatures.

Chloroplasts are essential organelles that determine how the photosynthesis of green plants perceive low temperature stress signals through membranes and photoreceptors. Previous studies have shown that plants experience a series of physiological and cellular regulatory activities under low temperatures, including alterations to calcium signaling, membrane structure and photosynthesis (Gan et al. 2019). Usually, the first type of metabolism to be affected is low temperature photosynthesis (Kočová et al. 2009). A rapid decline in growth at low temperatures severely inhibits photosynthesis in a number of plant species (Goulas et al. 2006). Under low temperature, the enzyme involved in the light reaction is more stable than that of the dark reaction, causing the photoinhibition of photosystem I and sometimes II and the production of reactive oxygen species (ROS) (Kenchanmane Raju et al. 2018). An excessive accumulation of ROS can damage the cell membrane system, produce malondialdehyde (MDA), cause electrolyte leakage in cells, and results in the increased electrical conductivity of plants (Farnsworth et al. 2012). Multiple types of antioxidant enzymes are expressed in response to low temperature to alleviate ROS damage (An et al. 2012; Cao et al. 2011; Kenchanmane Raju et al. 2018; Wen et al. 2019). It has been reported that cold-resistant species have more efficient antioxidant systems than sensitive species to protect the plants from ROS (Kočová et al. 2009). In addition, photosynthesis can also be enhanced by regulation of the abundance of photosynthesis-related proteins and the accumulation of starch grains and unsaturated fatty acids in chloroplasts under low temperature stress, thereby improving the cold resistance of plants (Gan et al. 2019; Popov and Astakhova 2021).

A proteomics analysis is a powerful tool to use to comprehend which proteins are present in specific tissue under abiotic stresses (Agrawal et al. 2015). In previous studies, proteomics analysis of cold-tolerant rice under low temperature treatment showed that 289 proteins changed significantly compared with the control and mainly involved in photosynthesis, metabolic pathway, biosynthesis of secondary metabolites and carbon metabolism (Chen et al., 2009). The photosynthesis, oxidative stress and cold acclimation were also identified as main pathways in response to cold according to 34 cold-responsive proteins in wild wheat (Gharechahi et al. 2014). In sugar beet, a total of 570 proteins were identified in vernalized and nonvernalized plants, of which the majority were assigned to phenylpropanoid biosynthesis, hormone metabolism and protein processing pathway (Liang et al. 2018). Several hundreds of proteins involved in photosynthesis and ROS homeostasis were also found in response to drought, salt stress and alkali stress in sugar beet (Yang et al. 2013; Wang et al. 2017; Geng et al. 2021; Liu et al. 2022). However, the response of the chloroplast proteome to low temperature has not been studied in sugar beet. In this study, we utilized label-free quantitative proteomic analysis to explore the changes in chloroplast proteome at low temperature. The identification of functional proteins related to low temperature resistance provides a theoretical basis to further understand the mechanisms of cold tolerance and learn to breed a variety of sugar beet that is resistance to cold.

## Materials and methods

### Plant materials and low temperature treatment

Seeds of sugar beet KWS9442, which has good chilling tolerance and resistance to rhizomania and root rot, were sown in pots that contained sterilized vermiculite. After germination, the seedlings were transferred to hydroponic culture in a Hoagland nutrient solution at pH 5.8 and grown in a growth chamber at 25 ± 2 °C, 140 µmol m^−2^ s^−1^ light intensity and a 14-h/10-h photoperiod. After 21 days, low temperature treatments (4 °C) were performed in a light incubator using the light conditions described above. The leaves of the sugar beet plants were sampled at 0, 3, 6, 12, 24, 36, 48, 72 and 120 h of low temperature treatment, after which they were flash-frozen in liquid nitrogen and stored at − 80 °C for further analysis. Three biological replicates were independently treated for each treatment.

### Chlorophyll content

The content of chlorophyll (Chl) was determined as described by Fargašová and Molnárová (2010). The leaf samples (0.2 g) were macerated and ground in 2–3 mL 95% ethanol. The tissues were grown further with 10 mL 95% ethanol until they became white. After standing for 5 min, it was filtered. The volume was fixed to 25 mL with 95% ethanol. The absorbance of the extract was then measured at 470 nm, 649 nm and 665 nm. The content of Chl content was calculated using the following formulae:

$${\text{C}}_{{\text{a}}} = 13.95{\text{A}}_{665} - 6.88{\text{A}}_{649}$$

$${\text{C}}_{{\text{b}}} = 24.96{\text{A}}_{649} - 7.32{\text{A}}_{665}$$

$${\text{Chl content }}\left( {{\text{mg}}/{\text{g}}} \right) \, = {\text{ C}}_{{\text{c}}} \times {\text{V}}/{\text{W}}$$

C_a_ and C_b_ C_c_ are the concentrations of chlorophyll a and chlorophyll b, respectively (mg/L); V is the total volume of the extract (mL), and W is the fresh weight of the sample (g).

### Relative electrical conductivity measurement

Leaf fragments (0.5 g) were sampled by hole puncher and placed in a test tube that contained 10 mL of deionized water. After shaking in a 25 °C water bath for 2 h, the conductivity of the solution was measured and recorded as Lt. After the determination, the test tubes were boiled for 20 min in a thermostatic water bath and cooled to 25 °C. The conductivity of extraction solution was determined after stirring and recorded as Lo. The relative electrical conductivity (REL) was calculated using the following formula: REL (%) = Lt/Lo × 100% (Bao et al. 2020).

### MDA content

The content of malondialdehyde (MDA) was determined using thiobarbituric acid (TBA) as previously described (Wang et al. 2018). The leaf samples (0.5 g) were homogenized in 5% TBA and centrifuged at 3000 g for 10 min. The supernatant was mixed with the same amount of 0.67% (w/v) TBA and cooled to room temperature after 30 min of boiling. The supernatant was centrifuged again at 3000*g* for 10 min. The absorbance at 450 nm, 532 nm and 600 nm was measured, and MDA content were calculated by the formulae:

$${\text{MDA}}\;{\text{content}}\;\left( {\mu {\text{mol}}/{\text{g}}} \right) = \left[ {6.45\left( {{\text{A}}_{532} - {\text{A}}_{600} } \right) - 0.56{\text{A}}_{450} } \right] \times {\text{V}} \div {\text{W}}$$

V is the volume of the extract solution (mL), and W is the fresh weight of the sample (g).

### SOD measurement

Seedling samples (0.5 g) were homogenized in 4 ml of 50 mmol/L phosphate buffer (pH 7.8) on the ice. The homogenate was centrifuged at 12,000*g* for 20 min at 4 °C, and the supernatant was used as the source of superoxide dismutase (SOD). The assay mixture (3 mL) contained 14.5 mM methionine, 3 mM EDTA-Na_2_, 2.25 mM NBT, 60 µM riboflavin and 40 μL of enzyme extract. The tubes were shaken while they were incubated for 20 min under a 40-W fluorescent lamp at 25 °C. The absorbance of the extract at 560 nm was measured with non-illuminated samples as the blanks. The SOD activity was expressed in units (50% NBT inhibition = 1 unit) min^−1^ g^−1^of tissue.

$${\text{SOD}}\;{\text{activity}}\;\left( {{\text{u}}/{\text{g}}\;{\text{FW}}} \right) = \left[ {\left( {{\text{Ack}} - {\text{AE}}} \right) \times {\text{V}}} \right]/\left( {1/2{\text{Ack}} \times {\text{W}} \times {\text{Vt}}} \right)$$

Ack and AE were the absorbance of the reference tube and sample tube, respectively; V was the total volume of sample solution (mL); Vt was the volume of enzyme solution for determination (mL), and W was the fresh weight of the sample (g).

### Screening of candidate *BvDREBs*

*AtDREBs* protein sequences were downloaded from the TAIR database (www.arabidopsis.org). Potential homologous genes were screened from the sugar beet proteome database (bvseq.boku.ac.at) using the BLASTP program with an E-value less than 10E^−5^ and a bit score > 100. Subsequently, a sequence alignment was performed between the *AtDREBs* gene and homologous genes using Cluster Omega (Boyce et al. 2015). The VT + F + I + G4 model was then selected based on the results of ModelFinder software. Finally, the phylogenetic trees were performed by Iqtree software (Minh et al. 2020) using maximum likelihood and visualized by EvolView (Zhang et al. 2012).

### Analysis of the pattern expression of *BvDREBs*

Total RNA was isolated from the young leaf tissues (100 mg) of sugar beet using RNA-easy Isolation Reagent (Vazyme, Nanjing, China) according to the manufacturer’s instructions. RNA concentrations and purity (OD_260/280_) were measured with a NanoDrop 2000c, and their integrity was assessed using 1% agarose gel electrophoresis. DNase I and PrimeScript RT reagent kits (Takara, Dalian, China) were used to eliminate the genomic DNA and prepare the first-strand cDNA, respectively. Quantitative real-time reverse phase–PCR (qRT-PCR) was performed in TB Green premix Ex Taq (Takara, Dalian, China) using the Mx3000P real-time PCR system with three biological replications based on previous experiments (Pi et al. 2020). The primers for *BvDREB*s were designed using Primer-BLAST, and *BvGAPDH* was used as an internal control (Additional file 3: Table S1). The relative levels of gene expression were calculated using the 2^−ΔΔCT^ method.

### Protein extraction and enzymolysis

The sugar beet chloroplast proteins were extracted from seedling leaves using a Minute Chloroplast Isolation Kit (Invent Biotechnologies, Plymouth, USA) following the manufacturer's instructions. Briefly, the leaves (200 mg) were submerged in buffer A in a microcentrifuge tube and pulverized with a plastic pestle. Pre-cooled buffer B was added to resuspend the precipitate after centrifugation. Then, the green precipitate of ready-for-use chloroplasts were collected. Protein extracts were lysed with SDT lysis buffer and separated by 12.5% SDS-PAGE. Protein enzymolysis was performed as reported by Coleman et al. (2017) using 300 μg protein per sample for filter-aided sample preparation and desalination on a C18 cartridge (Coleman et al. 2017). The filtrate was subjected to freeze-dry vacuuming and redissolved in an aqueous solution of 0.1% formic acid (FA). Part of the filtrate was subjected to high performance liquid chromatography-mass spectrometry (LC–MS/MS) analysis.

## LC–MS/MS measurement and analysis

Nanoflow reversed-phase LC separation was conducted on an EASY-nLC 1200 system. The mobile phase was composed of solvent A (99.9% H_2_O and 0.1 FA) and solvent B (14.9% H_2_O, 85% acetonitrile, and 0.1% FA). The LC separation was conducted using the following gradient: solvent B was started at 5% for 2 min at a flow rate of 300 nL/min and then increased to 40% over 100 min. Solvent B was subsequently rapidly increased to 100% in 8 min and maintained for 12 min before 100% solvent A was used for column equilibration. Electrospray MS and MS data were acquired on a Q-Exactive HF-X mass spectrometer. All the analyses were performed in the positive ion mode using a nano-electrospray ion source. Full scan MS spectra (m/z 300–1800) were acquired at 60,000 resolution and an automatic gain control target value of 3 × 10^6^ charges. For the top 20 precursor ions, high-resolution MS2 spectra were acquired in the Orbitrap with a maximum injection time of 50 ms at 15,000 resolution (isolation window 1.6 m/z), an AGC target value of 1 × 10^5^ and normalized collision energy of 28%.

## Proteomic data processing

The raw MS files were processed by MaxQuant and searched against the protein database (Beta_vulgaris.RefBeet-1.2.2.pep.all). Trypsin was set as the primary digest reagent, and carbamidomethyl as a fixed modification. The maximum number of missed cleavages was set to 2. Both peptides and proteins were filtered to a specified false discovery rate (FDR) < 0.01. It was to take 2 or 3 data from three biological repeated samples for data analysis. Proteins with the absolute value of the log2Ratio (24 h/ck) > 1.5 and *P*-values < 0.05 were considered to be significant differentially expressed proteins (DEPs). Finally, eulerAPE was used to compare the DEPs and draw the Venn diagram (Micallef and Rodgers 2014). To determine which proteins are transcribed and translated by chloroplast DNA, all of the protein sequences identified by MS were searched against the chloroplast protein database using BLASTP, and the threshold of E-value and score were set as E-value < 10E^−5^ and score > 50 (Stadermann et al. 2015).

## GO enrichment analysis of DEPs

Gene Ontology (GO) enrichment analysis was performed using AgriGO with default values (Du et al. 2010). References for the proteome annotation of beet were downloaded from PLAZA 4.0 Dicots (https://bioinformatics.psb.ugent.be/plaza/versions/plaza_v4_dicots/).

## Results

### Phenotype and physiological responses to low temperature stress in sugar beet

**Fig. 1:**
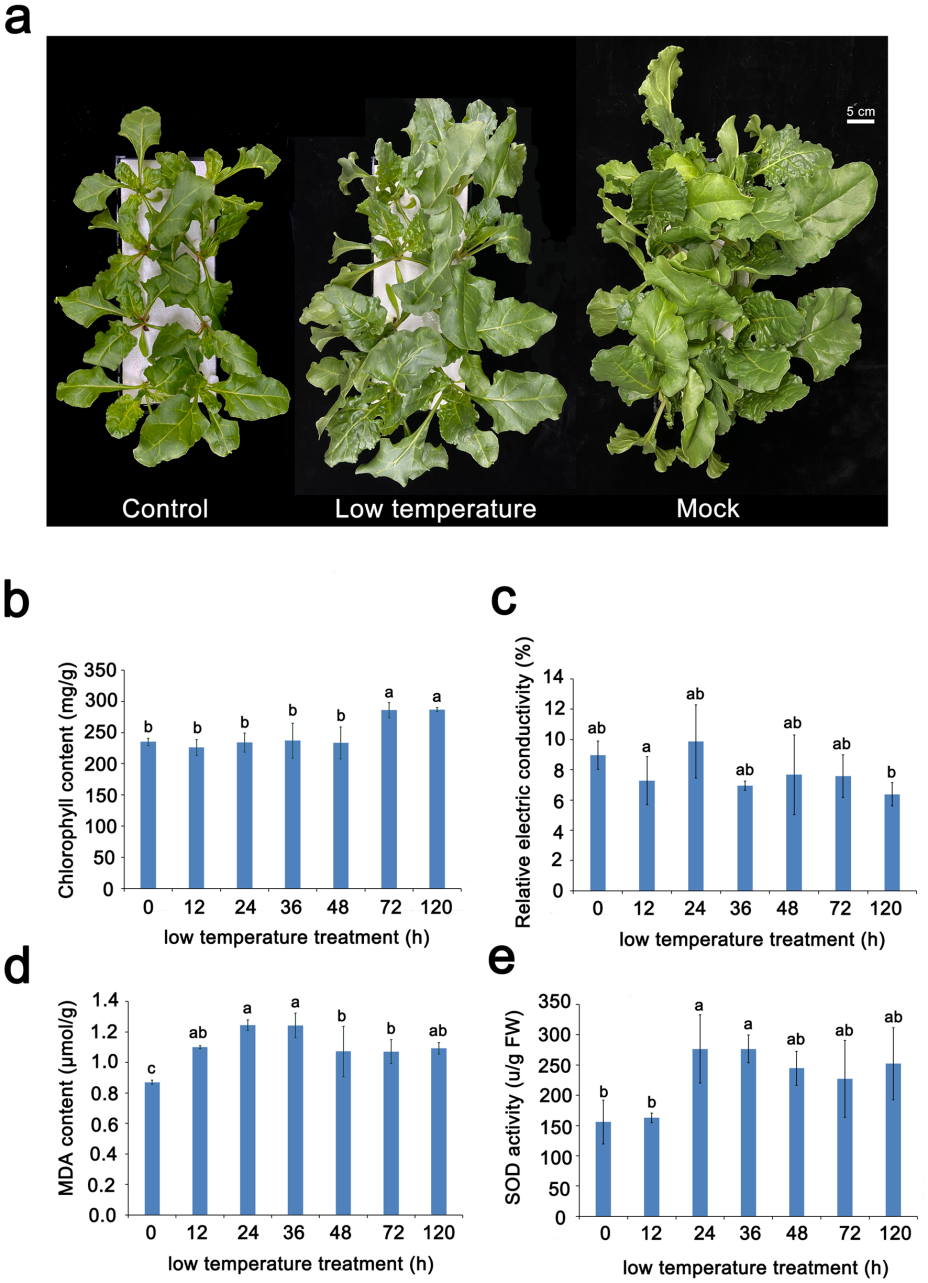
Morphological and physiological changes of sugar beet. **a** The phenotype of sugar beet treated with and without low temperature (4 °C). The graph of control was taken at 0 h after chilling. The graph of low temperature was taken at 120 h after chilling. The graph of mock was taken at 120 h without chilling. **b** Effect of low temperature (4 °C) on chlorophyll, **c** relative electrical conductivity (REL), **d** malondialdehyde (MDA), **e** superoxide dismutase (SOD) in sugar beet. Lowercase letters represent the significant level of P < 0.05 between different treatments

After 120 h of low temperature treatment, there were not obviously wilting, necrotic areas and external discoloration detected in leaves (Fig. 1a). However, low temperature treatment caused obvious growth inhibition compared with mock treatment, which sugar beet grew at room temperature for 120 h. Compared with the controls, there was no significant change in the Chl content within 48 h of low temperature treatment. After 72 h of low temperature treatment, the Chl content increased significantly to 20% (Fig. 1b). No significant change occurred in the damage of plasma membranes with REL of all the samples compared with the controls (Fig. 1c). However, a significant increase of MDA content was observed after 12 h of low temperature treatment (Fig. 1d). The activity of SOD increased and then reached its highest level after 24 h (Fig. 1e). These findings suggest that sugar beet responded positively within 24 h of low temperature treatment and was not significantly damaged in chloroplast.

## Analysis of the expression pattern of *BvDREBs* genes

**Fig. 2:**
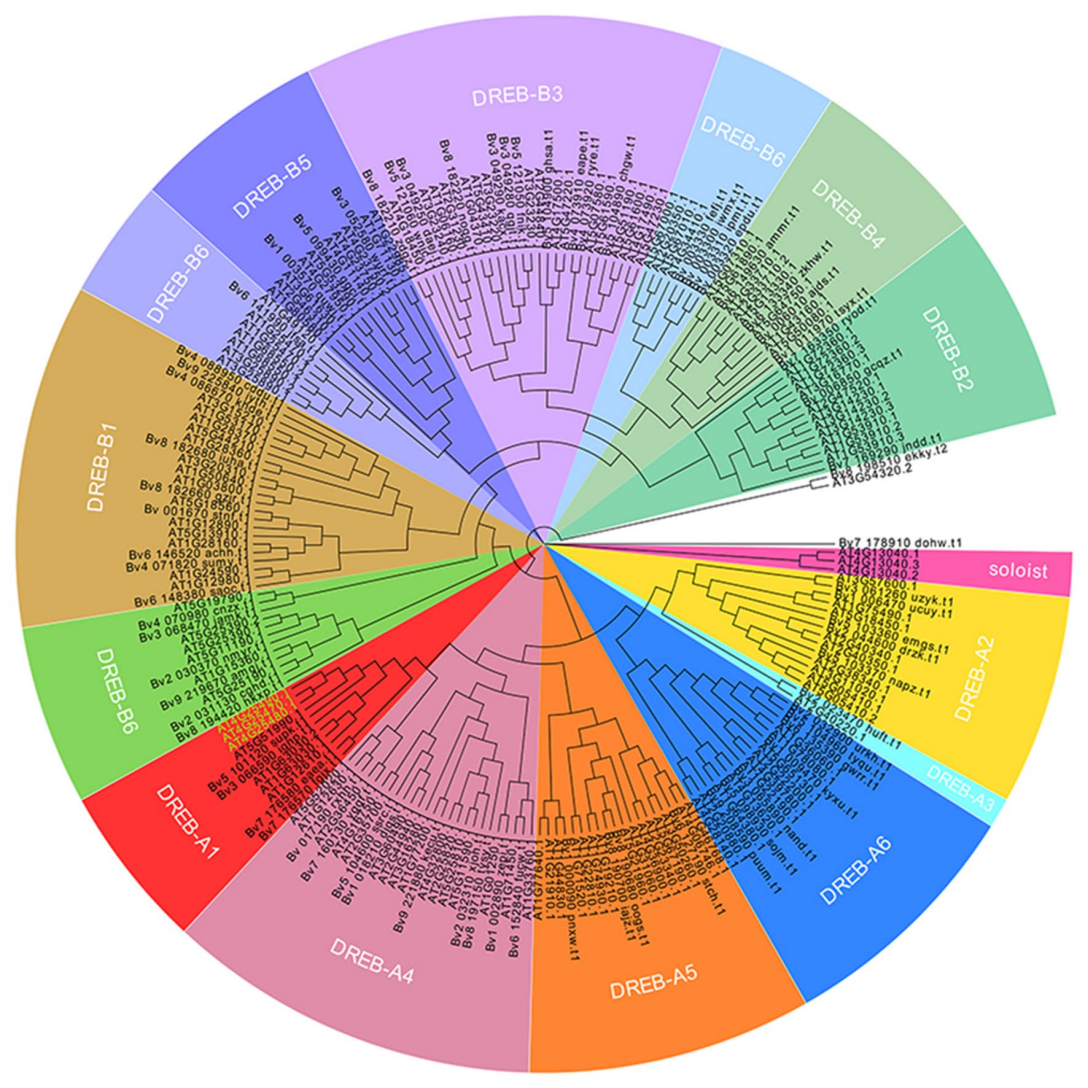
Phylogenetic analysis of the DREB family in sugar beet. Different background colors indicate different subfamilies, while the green letters in DREB-A1 are *AtDREB1A*, *AtDREB1B* and *AtDREB1C*, respectively

To profile the transcriptional response, *BvDREBs*, orthologous genes in *Arabidopsis thaliana* that act essential regulators in cold response pathway, were identified and selected as marker genes (Bo et al. 2007). A total of 73 homologous genes were identified in sugar beet using bidirectional BLAST searches. A phylogenetic analysis of these 73 homologous genes showed that 15 clades that contained the DREB-A1, DREB-A4 and soloist subfamilies were resolved (Fig. 2). The DREB-A1 subfamily played an important role in low temperature stress. As physiological drought is often accompanied by low temperature, the adjacent DREB-A4 subfamily that responded to drought was also considered as candidate genes.

**Fig. 3:**
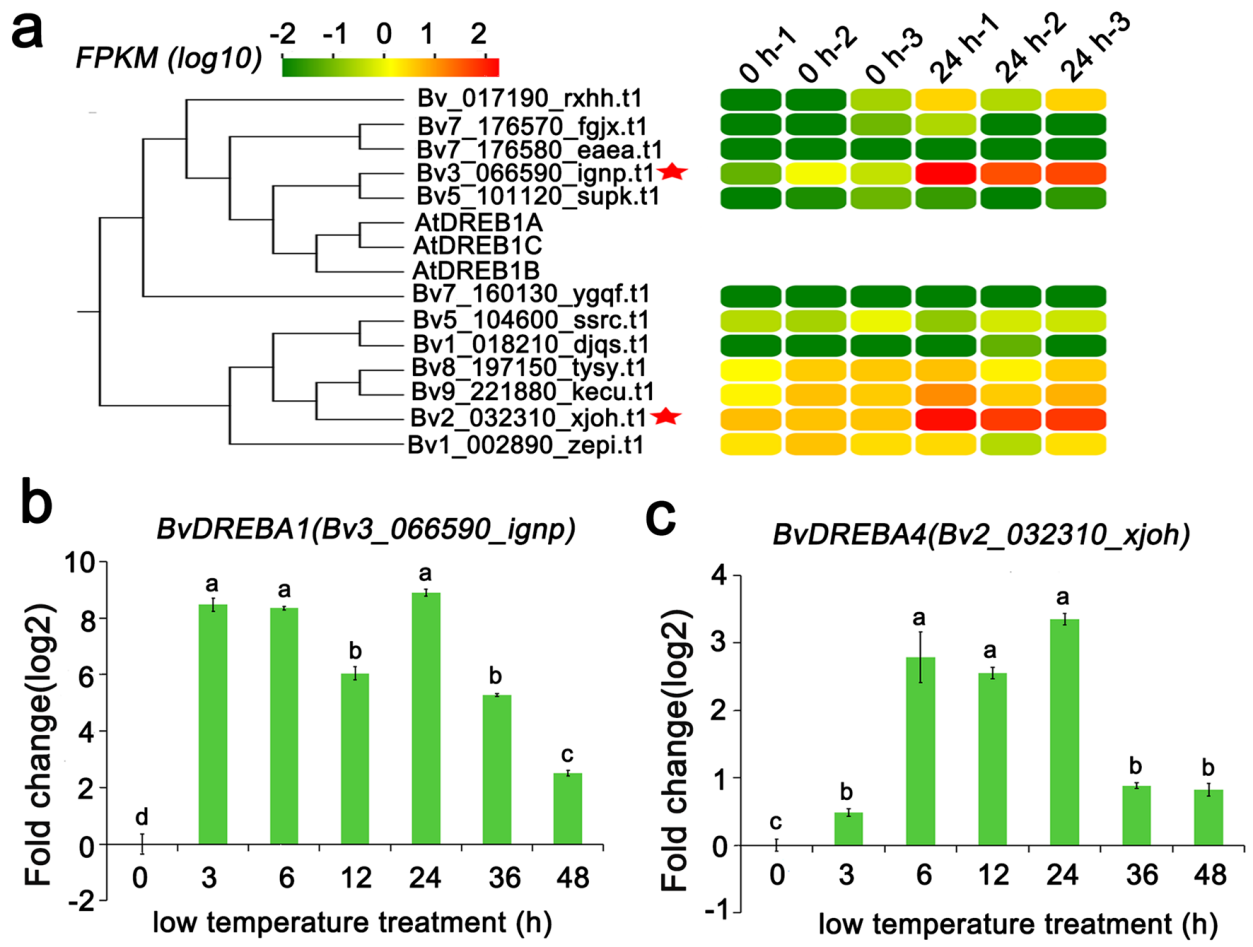
The patterns of expression of *BvDREBA1* and *BvDREBA4*. **a** Transcriptome analysis of gene expression of *BvDREBA1* and *BvDREBA4* at low temperature (4 °C). **b** The pattern of expression of *BvDREBA1* in response to low temperature (4 °C). **c** The pattern of expression of *BvDREBA4* in response to low temperature (4 °C). Different letters represent significant levels of P < 0.05 between different low temperature treatments

There were four sugar beet genes in the same branch as that of the *AtDREB-A1* gene. However, only *Bv3_066590_ignp* (*BvDREBA1*) was significantly up-regulated in four candidate genes after low temperature treatment based on the transcriptome data. Among of DREB-A4 subfamily genes, the *Bv2_032310_xjoh* (*BvDREBA4*) gene also responds to low temperature (Fig. 3a). Subsequently, the dynamic changes of expression of *BvDREBA1* and *BvDREBA4* were measured by qRT-PCR. The results indicated that *BvDREBA1* and *BvDREBA4* changed significantly within 3 h of the low temperature treatment and reached their peak after 24 h of treatment with low temperature. The level of expression of *BvDREBA1* and *BvDREBA4* began to gradually decease, and the level of expression after 48 h of low temperature treatment was significantly lower than that after 24 h of low temperature treatment (Fig. 3b, c).

## Chloroplast proteome identification

**Fig. 4:**
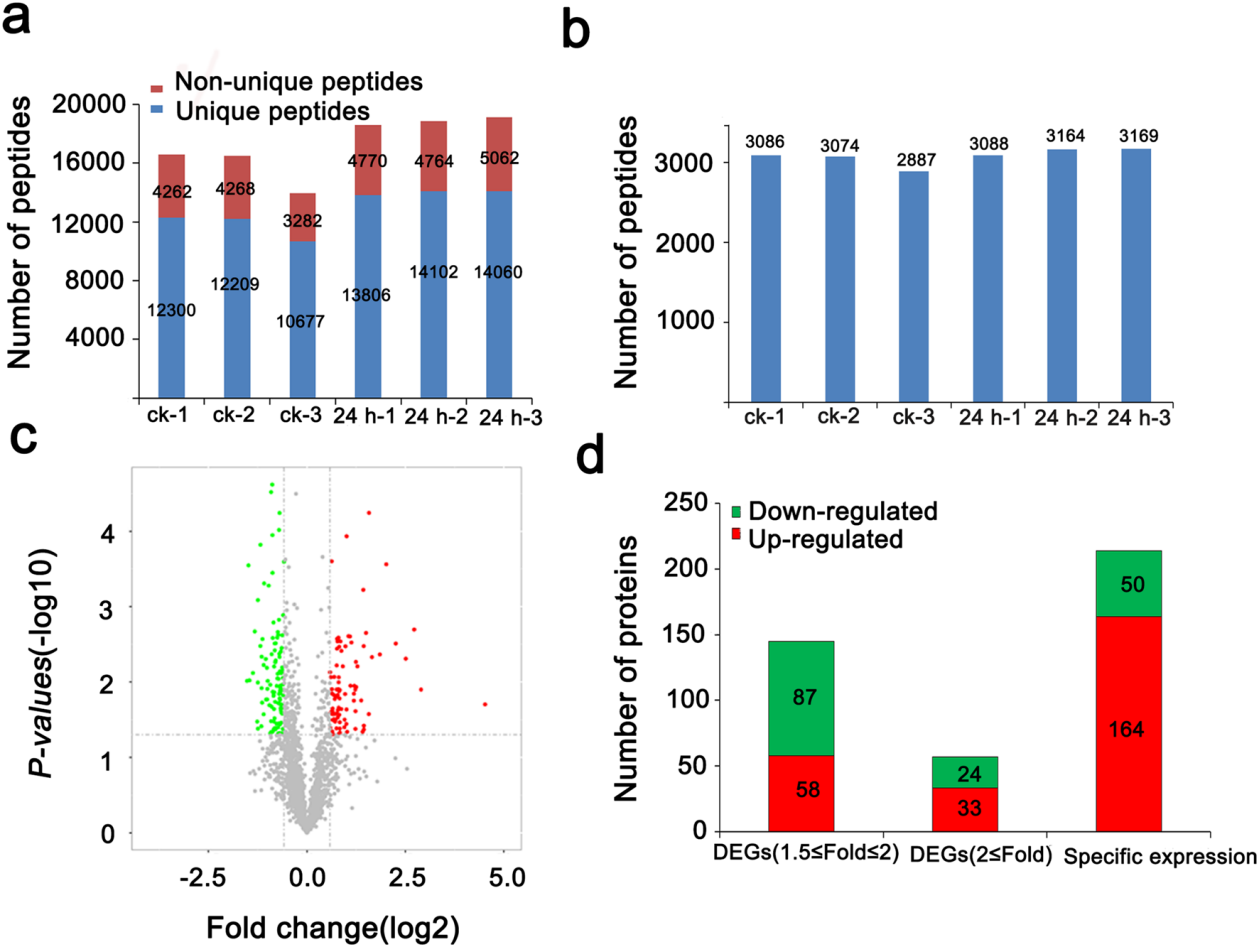
Statistics of protein identification results. **a** The number of peptides identified by LC–MS/MS. **b** The number of proteins identified by LC–MS/MS. **c** The volcano plot of the proteins identified in the CK and treatment. **d** The number of up- and down-regulated proteins

Based on physiological and transcriptional changes, sugar beets that had been treated by 24 h of low temperature, were selected as the samples to detect chloroplast proteomes in subsequent studies. After extraction of chloroplast protein (CK, 24 h), only protein samples with a highly abundant Ribulose-1,5-bisphosphate carboxylase/oxygenase (RuBisCo), as characteristic protein in chloroplasts, observed near 60 kDa and without obvious protein degradation was used for subsequent MS analysis. A total of 103,562 peptides (CK, 24 h) were identified by LC–MS/MS, with 16,423 unique peptides that corresponded to 3420 proteins. Moreover, it was notable that approximately 10,677–14,102 unique peptides that corresponded to 2887–3169 proteins were identified in each sample (Fig. 4a, b). The Pearson correlation coefficients between three biological repeats were > 0.97 in each group (Additional file 1: Fig. S1a). The log2 values of peptide intensity in each sample ranged from 25 to 30, and the peptides with different signal intensities had a normal distribution (Additional file 1: Fig. S1b). That finding demonstrated that not only are the biological samples in the group reproducible but also that the MS is highly stable.

A *t*-test was performed to investigate DEPs in response to low temperatures. The ratio fold changes log2(24 h/ck) > 1.5 and p-values < 0.05 were regarded as the thresholds for screening the DEPs between the CK and 24 h (Fig. 4c). A total of 416 DEPs were identified, including 91 up-regulated proteins and 111 down-regulated proteins, and 214 proteins were specifically expressed in the CK or 24 h (Fig. 4d, Additional file 4: Table S2). An analysis of the specific expression proteins indicated that 164 up-regulated and 50 down-regulated DEPs were detected. Among the up-regulated and down-regulated proteins, the expression of 33 and 24 proteins changed by more than two-fold, respectively.

## Identification of proteins encoding from chloroplast DNA

According to the sugar beet chloroplast genome (Stadermann et al. 2015), a total of nine proteins (0.3%) encoded by the chloroplast genome were identified by BLASTP (Additional file 2: Fig. S2a). They were primarily divided into three types, namely ribosomal proteins (5), RNA polymerases (3) and an ATP synthase (1) (Additional file 2: Fig. S2b). Among them, there were three DEPs, including two ribosomal proteins and one ATP synthase, accounting for 0.7% of the DEPs (Additional file 2: Fig. S2c, d). Additionally, the expression of these proteins has a high fold change in response to low temperature treatment. At 24 h of low temperature treatment, the ATP synthase protein was up-regulated 23-fold, and the protein of two ribosomes was up-regulated six-fold and two-fold, respectively. Large changes in protein abundance suggest that chloroplast DNA-encoded proteins also play an important role in response to low temperature or cold acclimation of sugar beet.

## GO enrichment analysis of DEPs

**Fig. 5:**
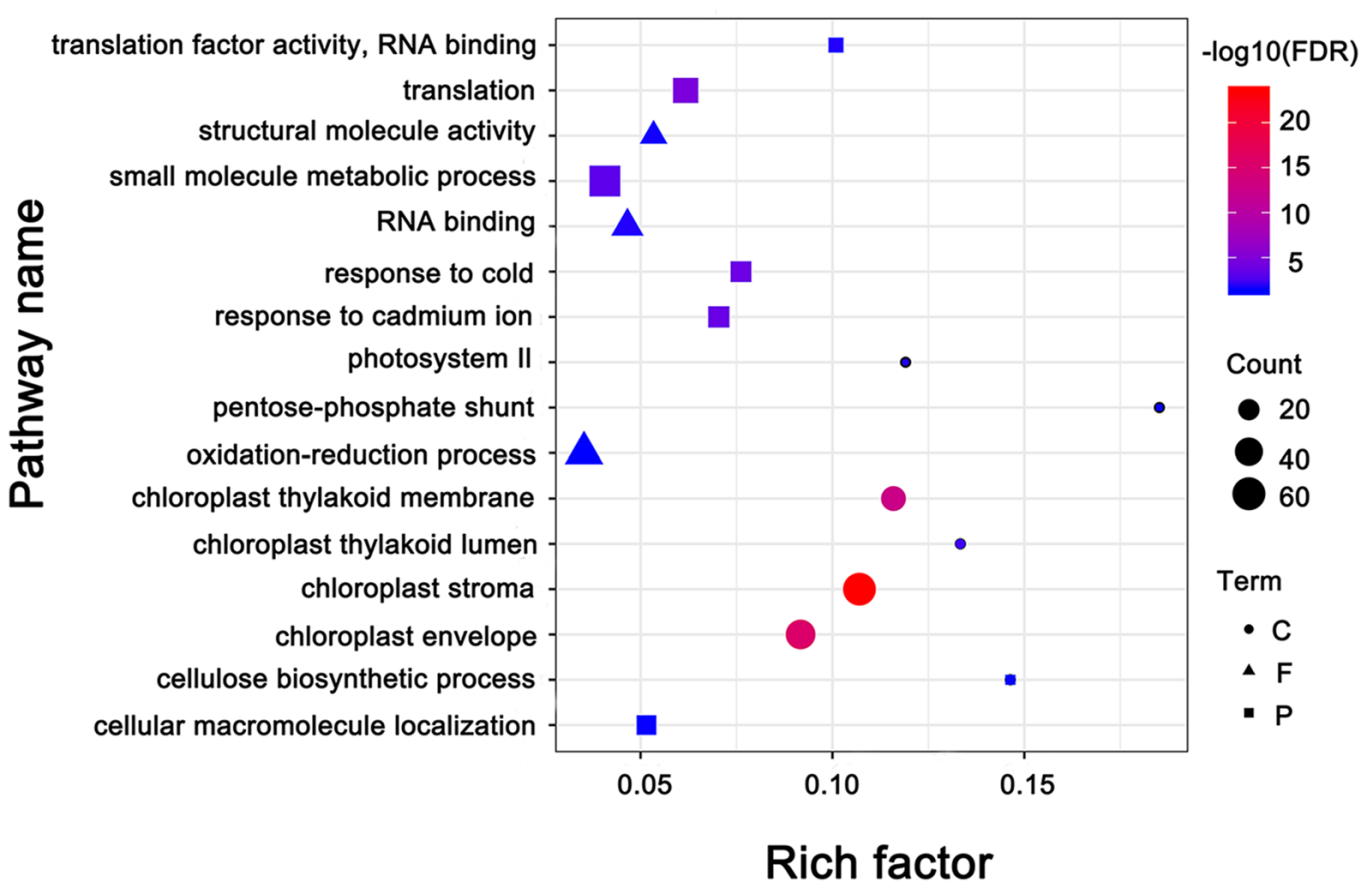
GO enrichment of significantly differentially expressed proteins

To explore the biological functions of the low temperature responsive proteins, we used AgriGO to analyze the DEPs at low temperature responsive stages. A total of 143 GO terms were significantly enriched, of these, including 47 biological process, three molecular function and 93 cellular components. Based on the relationship between the directed acycline praph and the false discovery rate (FDR), we screened 16 significantly enriched GO terms that play a major role in response to the low temperature of chloroplasts in sugar beets (Fig. 5).

There were eight significantly enriched GO terms in the biological process, among them, the most significantly enriched GO term was translation (GO:0006412), and its FDR value was 2.1E^−5^. Response to cold (GO:0009409), response to cadmium ion (GO:0046686) and small molecule metabolic process (GO:0044281) followed with FDR values of 1.4E^−4^, 2.2E^−4^ and 6.8E^−4^, respectively. Significantly enriched GO terms reflect that sugar beet chloroplasts initiate a response to low temperature through Ca^2+^ signaling and translate more novel proteins that are involved in the resistance to cold. RNA binding (GO:0003723), translation factor activity (GO:0008135) and structural molecule activity (GO:0005198) were significantly enriched in the molecular function, with FDR values of 0.022, 0.022 and 0.033, respectively. Five GO terms in the cell component were significantly enriched. The FDR values of chloroplast stroma (GO:0009570), chloroplast envelope (GO:0009941) and chloroplast thylakoid membrane (GO:0009535) were 3.3E^−24^, 3.1E^−16^ and 2.3E^−13^, respectively, which were the most significant in all of the GO terms. This finding indicates that the chloroplast stroma, chloroplast envelope and chloroplast thylakoid membrane are the primary components of low temperature response.

**Fig. 6:**
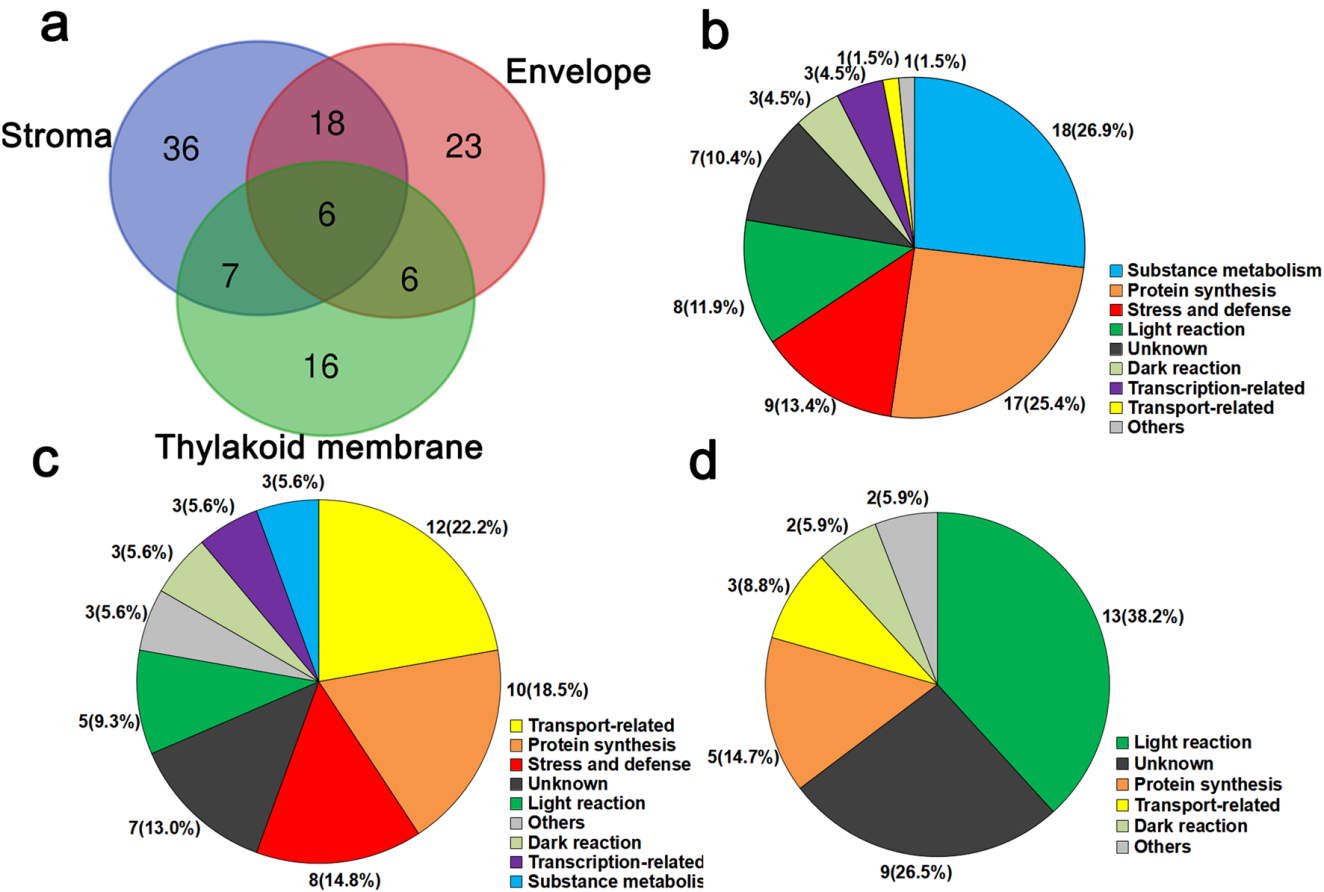
Functional annotation of differential expressed proteins in different subchloroplast fractions. **a** Venn diagram. **b** Chloroplast stroma. **c** Chloroplast envelope. **d** Chloroplast thylakoid membrane

The functions of the DEPs located on chloroplast components were classified in more detail. A total of 112 DEPs were involved in the low temperature response in chloroplast stroma (GO:0009570), chloroplast envelope (GO:0009941) and chloroplast thylakoid membrane (GO:0009535) (Fig. 6a).

The number of proteins related to substance metabolism and protein synthesis was the largest in the chloroplast stroma (GO:0009570), with 18 and 17, accounting for 26.9 and 25.4%, respectively (Fig. 6b). Five proteins were up-regulated, and 13 proteins were down-regulated in substance metabolism. These types of substance metabolism involved the pentose phosphate pathway, starch metabolism and synthesis. In addition, the DEPs related to protein synthesis primarily included the 50S large subunit and 30S small subunit that form ribosomes, and protein translation and folding related proteins, such as tRNA aminotransferase, translation elongation factor and peptidyl prolyl cis–trans isomerase. Four ribosomal proteins significantly increased after 24 h of low temperature treatment. Moreover, there were also a large number of proteins related to stress defense (9) and light reactions (8), accounting for 13.4% and 11.9%, respectively. In stress and defense, Cu/Zn-SOD, CAT, peroxiredoxin (Prx) and thioredoxin (Trx) were involved to activate the H_2_O_2_ signaling-related proteins and ROS scavenging.

The number of transport-related proteins was the largest chloroplast envelope in (GO:0009941). They totaled 12, accounting for 22.2%, primarily including Toc-Tic complexes, glucose transporters, aquaporins, and ADP/ATP transport among others (Fig. 6c). Protein synthesis followed with 10 DEPs, accounting for 18.5%, including the 50S large subunit and 30S small subunit. There were seven unknown proteins accounting for 13%, which could be related to the low temperature response. The role of these proteins in low temperature response merit additional study in the future.

The main low temperature responsive proteins in chloroplast thylakoid membrane (GO:0009535) were concentrated in the light reaction and included 13 proteins, accounting for 38.2% (Fig. 6d). These proteins are involved photosystem I (PS I), photosystem II (PS II), quinone oxidoreductase, and ATP synthase. Additionally, nine unknown proteins still responded to low temperature stress, accounting for 26.5%. The function and relationship with the light reaction still merit further study.

## Molecular mechanisms of the chloroplast that underlie low temperature in sugar beet

**Fig. 7:**
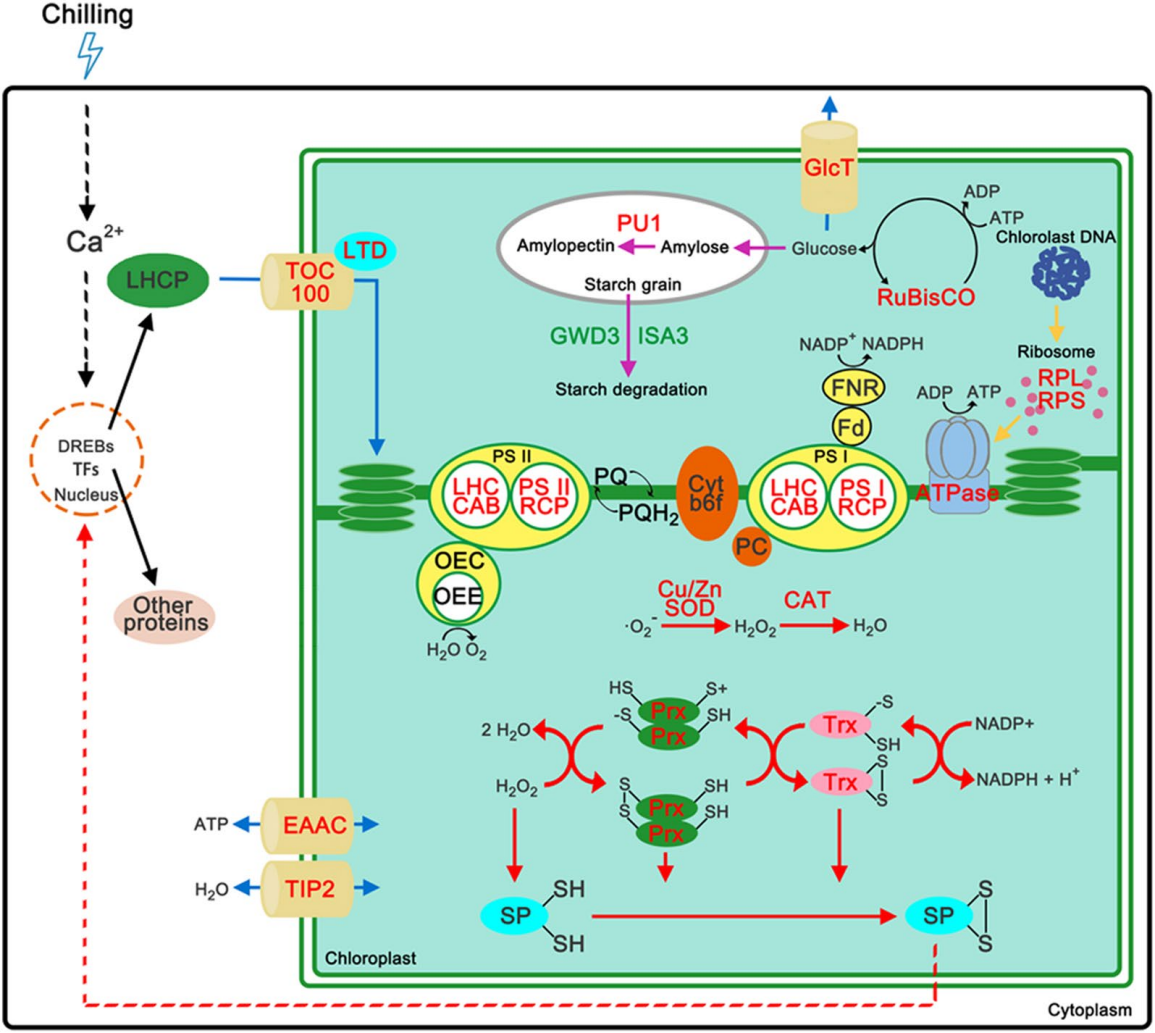
Schematic presentation of systematic chilling response mechanisms in chloroplast of sugar beet. Red and green letters represent proteins were significantly up- and down-regulated after chilling, respectively. Blue, red, yellow and purple arrows represent transport-related pathway, antioxidant pathway and H_2_O_2_ signal, translation from chloroplast DNA and starch metabolism, respectively. The dotted line represents the signal transduction pathway

This analysis revealed the following processes that sugar beets use to manage low temperature tolerance in chloroplast of sugar beet (Fig. 7). After low temperature treatment for 24 h, a large number of proteins significantly increased in the photosynthetic system. Notably, most photosynthetic proteins were expressed in the nuclear genomes and then entered the chloroplasts through the Toc-Tic complex, including BvTOC100, under the control of BvLTD. In additional, chloroplast genes were also induced by low temperature to initiate transcription and translation. ATP synthase was highly expressed under the induction of chloroplast ribosomal protein. The α subunit in particular was up-regulated 23-fold. An increased abundance of these proteins may lead to an increase in glucose content in chloroplasts. These glucose molecules can be used as osmotic regulators by transportation outside of the chloroplasts via BvGlcT. Moreover, it can also be synthesized as amylose. Amylose is further metabolized to amylopectin under the action of enzymes, such as BvPU1. The abundance of starch degradation-related proteins (BvISA3 and BvGWD3) decreased significantly, indicating that low temperature could induce the formation of starch granules in sugar beet chloroplasts. Finally, to protect the ROS generated through photosynthetic electron transport, the ROS scavenging system composed of BvCu/Zn-SOD and BvCAT was also induced by low temperature. The up-regulation of BvPrx and BvTrx protein not only enhanced the removal of H_2_O_2_ but were also involved in the oxidation of signaling proteins, such as transcription factors and phosphatase, enabling signaling via the transmission of H_2_O_2_ to the nucleus and up-regulating the expression of stress and defense-related proteins.

## Discussion

The chloroplast is the main place of plant photosynthesis and is also very sensitive to ambient temperature. Low temperature stress can significantly inhibit the activity of chloroplast proteins, interfere with photosynthetic electron transport and photosynthetic phosphorylation, cause the accumulation of ROS, and finally cause oxidative damage (Calzadilla et al. 2019). Relatively, short-term or intermittent low temperature stress increases the resistance of plants to low temperature, a phenomenon known as cold acclimation (Gerber et al. 2021). In *A. thaliana*, *AtDREBs* are important transcription factors that regulate cold acclimation and are involved in gene activation and the inhibition of Ca^2+^ and hormone signals and carbon metabolism, respectively (Zhao et al. 2016). The results of this study showed that low temperatures could induce the expression of *BvDREB* genes, reaching the highest level after 24 h, suggesting that cold acclimation was activated. To explore which proteins were involved in the cold acclimation of chloroplasts in sugar beet, the proteome was detected using the Label-free technique before and after treatment with low temperature. A total of 416 proteins was found to be significantly affected by low temperature. An AgriGO analysis showed that proteins from the chloroplast thylakoid membrane, envelope and stroma were the most sensitive to low temperature. In these compartments, the proteins involved in photosynthesis, starch metabolism, chloroplast protein translation, protein transport and antioxidant processes should be closely related to the cold acclimation of sugar beet.

## Effect of low temperature on chloroplast thylakoid membrane proteins in sugar beet

The chloroplast thylakoid membrane is the main site for light reaction. Different crops were subjected to short-term low temperature treatment, and the changes in the protein abundance of photosynthetic system differed substantially. In wheat, the expression of 33 kDa oxygen evolving complexes, oxygen-evolving enhancer protein and light-harvesting complex protein were significantly repressed by low temperature treatment, while the expression of light-harvesting chlorophyll a/b binding protein was significantly up-regulated (Rinalducci et al. 2011; Herman et al. 2006). Interestingly, low temperature treatment in rice and barley resulted in a completely opposite expression profile for these proteins (Rinalducci et al. 2011; Hashimoto and Komatsu 2007). In this study, a total of 13 proteins involved in the light reaction were found to be significantly up-regulated after low temperature treatment for 24 h. Seven proteins changed more than two-fold, and two proteins were specifically expressed at low temperature treatment for 24 h. These proteins involved multiple components of the light reaction, such as PS I, PS II, plastoquinone, and ATP synthase. In particular, the ATP synthase α subunit was up-regulated to 23-fold. A low temperature-induced protein that is involved in the light reaction could help to increase the supply of energy during photosynthesis and provide the necessary energy for the cold acclimation of sugar beet. In addition, it will help to maintain the balance between capability of light capture and consumption at continuous low temperatures. Similarly, after 2 weeks of cold acclimation at 4 °C, the chlorophyll fluorescence parameter Fv/Fm of the *A. thaliana* seedlings remained > 0.8, which was similar to the ratio at room temperature (Buer et al. 2016). The detection of various photosynthetic parameters showed that the net photosynthetic rate, stomatal conductance and chlorophyll fluorescence parameter were less affected during cold acclimation in winter wheat (Li et al. 2014).

## Effect of low temperature on chloroplast stroma proteins in sugar beet

Chloroplast DNA primarily exists in the chloroplast stroma that contains the proteins involved in transcription and translation of chloroplast genomes. In addition, the region is also the main place of carbon assimilation and alkaloid metabolism. These proteins primarily participated in carbon metabolic pathways, such as the Calvin cycle, glycolysis, pentose metabolism and chloroplast protein synthesis and assembly process, based on a two-dimensional electrophoresis analysis of the chloroplast stroma proteome in *A. thaliana* (Peltier et al. 2006). Changes in the chloroplast proteome were analyzed in *A. thaliana* after low temperature treatment 24 h using DIGE techniques. The results indicated that 43 proteins are significantly expressed in chloroplast stroma, primarily RuBisCo, glyceraldehyde-3-phosphate dehydrogenase and 2-Cys antioxidant proteins (Goulas et al. 2006). Consistent with the results of this study, RuBisCo and 2-Cys antioxidant proteins were also found to be induced by low temperature. Glucose-6-phosphate dehydrogenase and 6-phosphogluconolactonase were also significantly down-regulated after treatment with low temperature, suggesting that the pentose phosphate pathway was inhibited by low temperatures in sugar beet chloroplasts. Moreover, the proteins involved in starch metabolism changed significantly at low temperature. α-Glucan and water dikinase (BvGWD3) can catalyze the phosphorylation of the C3-position of starch, change the surface structure of starch granules, and further promote the cleavage of the glycosidic bond (Orzechowski et al. 2013). Isoamylase (BvISA3) can specifically recognize the α-1,6-glycosidic bond of amylopectin, thus promoting starch hydrolysis by causing the debranching of amylopectin (Delatte et al. 2006). Pullulanase (BvPU1), similar to isoamylase, can specifically recognize and hydrolyze the α-1,6-glycosidic linkages in amylopectin. Unlike BvISA3, BvPU1 regulates the synthesis of amylopectin by catalyzing the hydrolysis of the terminal branches of the amylopectin precursor (Streb et al. 2008). BvGWD3 and BvISA3 proteins were significantly down-regulated after low temperature treatment in the chloroplasts of sugar beet, while BvPU1 was specifically expressed. These results suggest that low temperature can inhibit the degradation of starch and induce the synthesis of amylopectin, therefore leading to the formation of starch grains in the chloroplast. The observation showed that a large amount of starch granules began to accumulate by transmission electron microscopy in the chloroplasts of paper mulberry (*Broussonetia papyrifera*) leaves after low temperature treatment for 24 h. The transcriptome and proteome analyses led to the hypothesis that the accumulation of starch granules is an important mechanism for maintaining the Calvin cycle and photorespiration-mediated redox homeostasis in paper mulberry under low temperature conditions (Peng et al. 2015). In protein synthesis, the totals of three 50S ribosomal proteins (BvRpl6, BvRpl21, and BvRpl31), one 30S ribosomal protein (BvRps17) and one 28 kDa ribosomal protein were found to be significantly up-regulated by low temperature, indicating that the transcription and translation of chloroplast DNA closely relates to a low temperature response. A reverse genetics study found that ribosomal protein Rpl33 is not a necessary for plant growth in tobacco (*Nicotiana tabacum* L.), but it is closely related to low temperature tolerance (Rogalski et al. 2008). The Fv/Fm of *rpl33* mutant decreased more quickly than that of wild type under low temperature stress, and the abundances of PsbD and PsaB proteins significantly decreased (Rogalski et al. 2008). In this study, a significant increase in ribosomal protein may contribute to promote the synthesis of photosystem proteins to form a dynamic balance with the damage to the proteins of photosystem and maintain normal photosynthesis in the chloroplasts of sugar beet under low temperature conditions.

## Effect of low temperature on the chloroplast envelope proteins in sugar beet

A chloroplast envelope is composed of two layers of membrane at the edge of chloroplast, which is an important area to determine the entry and exit of water, ions, carbohydrates, proteins and other substances in the cytoplasm. In this study, 12 membrane transporters, including aquaporins, glucose transporters, ATP-binding cassette transporters and Toc-Tic complexes, were detected. These proteins are widely involved in the transport of carbohydrate, water, ATP, protein and other substance. The abundance changes in transporters will contribute to the establishment of new homeostasis in chloroplasts under low temperature conditions. Typically, the chloroplast proteome includes > 3000 proteins, of which only 3–5% (80–100) proteins were derived from chloroplast genes (Fristedt 2017). Therefore, most chloroplast proteins need to cross the chloroplast envelope to be transported into chloroplasts to perform their functions after translation in the nuclear genome. The Toc-Tic complex is usually considered to be the primary transporter of chloroplast proteins. BvTIC100 were significantly up-regulated after 24 h of low temperature treatment. In Arabidopsis, AtTIC100 interacting with AtTIC214, AtTIC56, and AtTIC20-I forms stable 1 MDa TIC complex transporting protein precursor for chloroplast biogenesis (Kikuchi et al. 2013). Moreover, BvLTD was specifically expressed and can be used as an anchor protein to participate in the process of light-harvesting chlorophyll-binding proteins into the chloroplast under low temperature and transfer these proteins to the chloroplast signal recognition particle-mediated protein recognition transport pathway (Ouyang et al. 2011). These results suggest that the chloroplasts of sugar beet preferentially import photosystem-related proteins into chloroplasts to maintain normal photosynthesis by increasing the abundance of anchored proteins under low temperature stress. This is consistent with the significant increase of proteins involved in the light reaction detected in this study.

## Effects of low temperature on antioxidant proteins in chloroplasts

The balance between light energy capture and light energy consumption can be destroyed by low temperature stress, and photosystem II overload leads to irreversible damage. In addition, low temperature stress can slow down the consumption of NADPH and accumulate electron acceptors in photosystem I (NADP^+^) by inhibiting enzyme activities in the Calvin cycle that eventually lead to electron transfer to oxygen molecules to generate ROS. The excessive accumulation of ROS can damage the cell membrane system, produce MDA and lead to the leakage of intracellular substances. The physiological responses of five wheat varieties to low temperature were measured, and the results showed that the contents of REL and MDA were higher during the overwintering period than those in the pre-wintering period (Zhang et al. 2016). After low temperature treatment at 8 °C for 4 days, the contents of both REL and MDA increased to more than two-fold of the original in cucumber leaves (Hu et al. 2006). Similar to the results of previous studies, the contents of REL and MDA also significantly increased in sugar beet leaves after low temperature (4 °C) treatment for 3 days and gradually increased with low temperature. These results showed that continuous low temperature can produce photoinhibition in sugar beet seedlings. To alleviate the oxidative damage caused by low temperatures, plants have evolved multiple antioxidant pathways. SOD can catalyze superoxide anion radicals to produce H_2_O_2_ and O_2_ that are primarily divided into three types based on their different auxiliary functions, namely Cu/Zn-SOD, Fe-SOD and Mn-SOD, and Cu/Zn-SOD is primarily distributed in chloroplasts (Han et al. 2020). BvCu/Zn-SOD proteins were significantly induced by low temperature. The H_2_O_2_ generated by SOD can be further degraded by CAT and peroxidase, so as to scavenge ROS. Similarly, BvCAT is also induced by low temperature. The ascorbic acid-glutathione cycle is considered to be the most important pathway for plants to scavenge ROS, which is widely found in chloroplasts, cytoplasm, mitochondria and peroxides. Water and monodehydroascorbic acid (MDHA) are produced by the reduction of ascorbic acid and hydrogen peroxide under the catalysis of ascorbic acid peroxidase, and some MDHAs are converted further into dehydroascorbic acid (DHA). Subsequently, MDHA generates ascorbic acid under the action of monodehydroascorbate reductase. In addition, DHA and GSH generate ascorbic acid and glutathione disulfide (GSSG) under the action of dehydroascorbate reductase. Finally, GSSG produces GSH owing to the activities of glutathione reductase (Wu et al. 2019). However, this study did not detect any significant changes in these proteins induced by low temperature. In contrast, thiol peroxidases (Prx) and thioredoxin (Trx), such as 1-Cys and 2-Cys peroxidases, were detected to be significantly up-regulated by low temperature. With the exception of the degradation of H_2_O_2_, Prx and Trx are involved in the oxidative modification of H_2_O_2_ signaling proteins in three ways (Netto and Antunes 2016). First, Prx can indirectly interfere with H_2_O_2_ and direct oxidation of the signal protein by regulating the concentration of intracellular H_2_O_2_. Secondly, Prx can catalyze the oxidation of signal proteins during the degradation of H_2_O_2_. Finally, the Prx oxidized by H_2_O_2_ makes a signal protein that is oxidized under the action of Trx (Netto and Antunes 2016). The results of this study suggest that the low temperature-induced expression of Prx and Trx can further induce the expression of resistance-related genes by activating the H_2_O_2_ signaling pathway in chloroplasts of sugar beet, and further enhance the cold acclimation.

## Conclusions

This study preliminarily explored the mechanisms of response of sugar beet chloroplasts and identified some important chloroplast proteins in response to low temperature stress. Four pathways of cold acclimation in the responses of sugar beet to low temperature were analyzed, which can be used to guide subsequent research. In this experiment, we found that the Toc-Tic complex could preferentially import photosynthesis-related proteins into chloroplasts under the control of BvLTD, so as to maintain the photosynthesis of sugar beet at low temperature. In addition, Prx and Trx can also be induced to activate the H_2_O_2_ pathway by low temperature, induce the expression of resistance genes, and enhance the cold acclimation of sugar beet. These two points can be used as the key contents of a follow-up study that could provide direction to screen cold-resistant genes in sugar beet and have important significance for the cold-resistant breeding of this crop.

## Supplementary Information

**Additional file 1: Table S1.** Primers used for qRT-PCR in this experiment.

**Additional file 2: Table S2.** The list of DEPs.

**Additional file 3: Figure S1.** Protein quality control analysis of different samples. **a** The correlation between the control and 24 h treatment. **b** Examination of peptide intensity distribution in different samples.

**Additional file 4: Figure S2.** **a** The number of proteins encoded from chloroplast DNA in this study. Ratio of proteins encoded by chloroplast DNA with those encoded by nuclear DNA. **b** The function of proteins encoding from chloroplast DNA. **c** Ratio of chloroplast DNA and nuclear DNA that encode differentially expressed proteins. **d** The function of different expression proteins encoding from chloroplast DNA.

## Data Availability

The data used and analyzed in this study can be provided from the corresponding author for scientific, non-profit purpose.

## Abbreviations

Chl: Chlorophyll
REL: Relative conductivity
MDA: Malondialdehyde
SOD: Superoxide dismutase
DREB: Dehydration responsive element binding protein
DEPs: Differentially expressed proteins
Prx: Peroxiredoxin
Trx: Thioredoxin
ROS: Reactive oxygen species
PBS: Phosphate-buffered saline
FA: Formic acid
LC–MS: Liquid chromatography-mass spectrometry
GO: Gene ontology
PS I: Photosystem I
PS II: Photosystem II
MDHA: Monodehydroascorbic acid
DHA: Dehydroascorbic acid
