# Supplementary figures and images for: Comparative proteomic analysis on chloroplast proteins provides new insights into the effects of low temperature in sugar beet
Source: Bot Stud. 2022 Jun 7;63:18. doi: 10.1186/s40529-022-00349-6 (PMC9174413; doi:10.1186/s40529-022-00349-6)

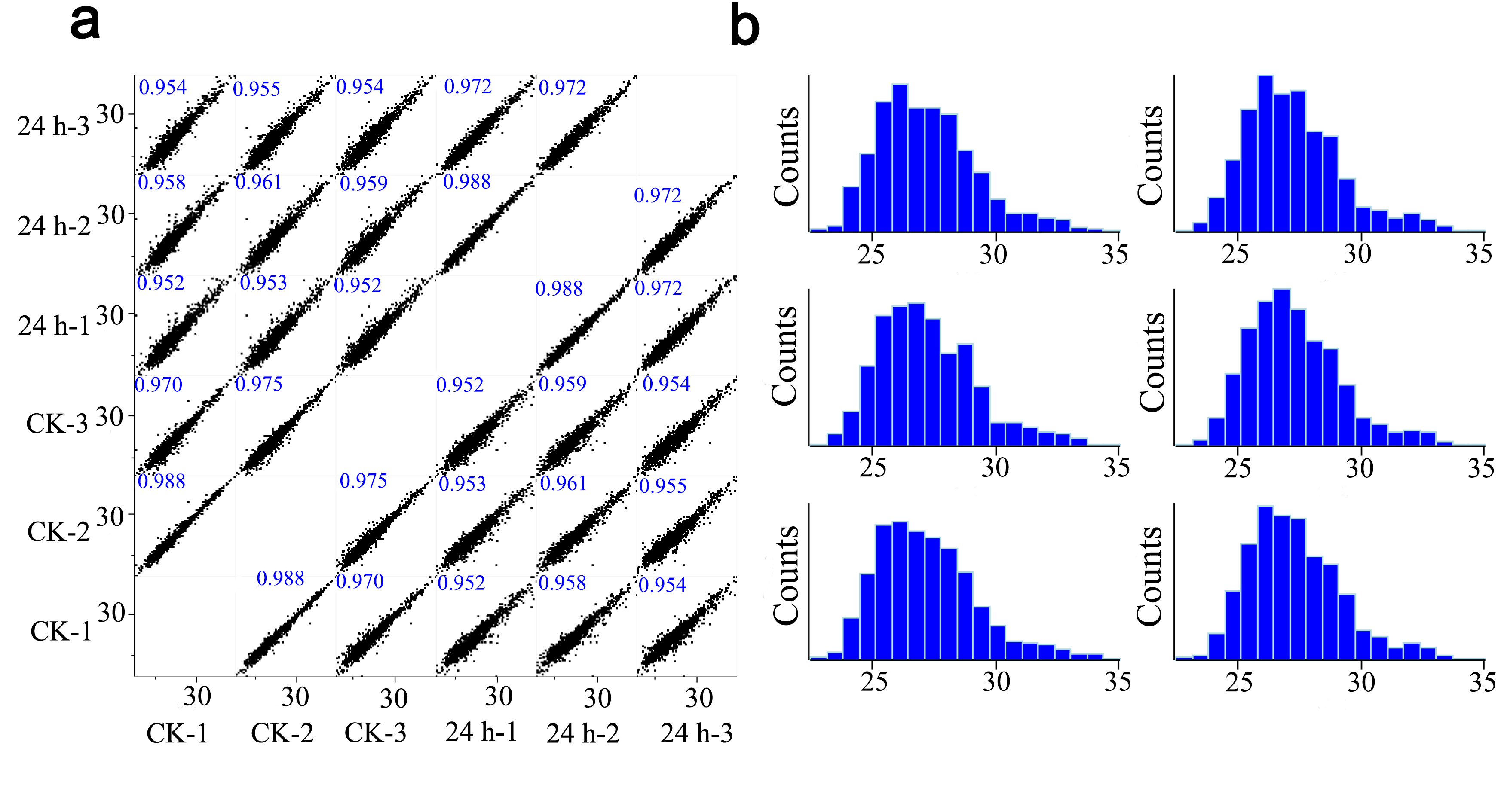

Supplement: Supplementary file 1 — Additional file 1: Table S1. Primers used for qRT-PCR in this experiment. [file 40529_2022_349_MOESM1_ESM.tif]

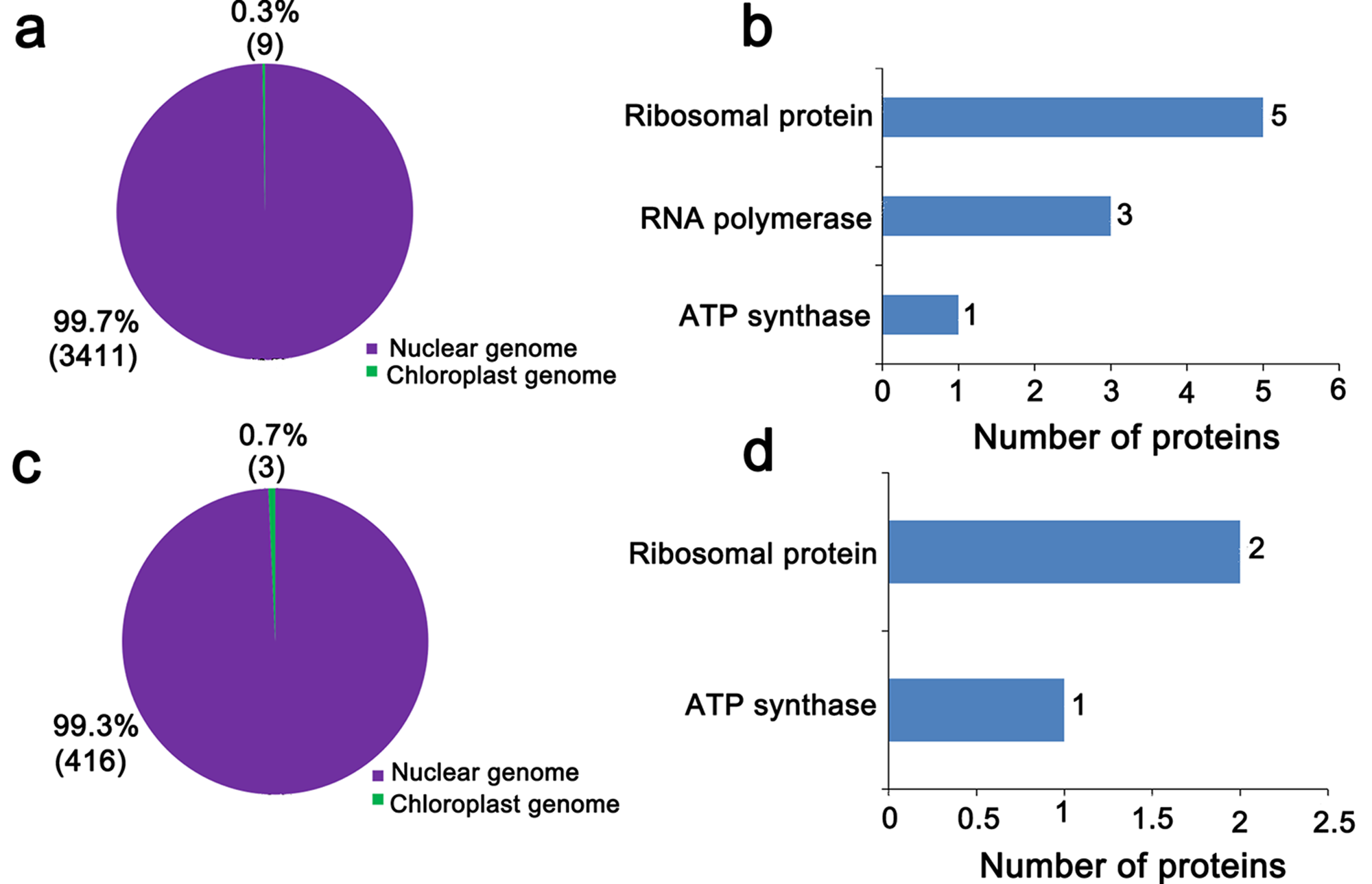

Supplement: Supplementary file 2 — Additional file 2: Table S2. The list of DEPs. [file 40529_2022_349_MOESM2_ESM.tif]
